# Gaps in Osteoporosis Management in a Predominantly Hispanic Population

**DOI:** 10.7759/cureus.96704

**Published:** 2025-11-12

**Authors:** Rachel L Moraes Dantas, Jenivia Sekar, Sudhagar Thangarasu, Gowri Renganathan

**Affiliations:** 1 Internal Medicine, Texas Tech University Health Sciences Center El Paso, El Paso, USA; 2 Internal Medicine, Florida International University, Miami, USA

**Keywords:** bone mineral density, chart review, hispanic population, osteopenia, osteoporosis, osteoporosis treatment gap

## Abstract

Introduction: Osteoporosis is a common disease with well-established guidelines for bone mineral density assessment and pharmacological treatment. However, it often remains underdiagnosed and untreated.

Objectives: This study aimed to describe osteoporosis risk factors, assess the use of the Fracture Risk Assessment Tool (FRAX), and evaluate the treatment gap among women with osteopenia or osteoporosis in the community served by our clinic.

Methods: The present study is a retrospective chart review of 387 women diagnosed with osteopenia/osteoporosis, who were seen at the Texas Tech University Health Sciences Center (TTUHSC) physician clinics in El Paso, over the course of one year (January 1, 2023-December 31, 2023). Inclusion criteria were medical charts with the ICD-10 (10th revision of the International Classification of Diseases) codes for osteoporosis and osteopenia (M81.0, M81.8, M80, and M85.80). Charts of women under 50 years of age and all men were excluded. Collected data includes demographics (self-reported, race/ethnicity and age at the time of diagnosis), previous medical history (previous fractures, smoking history, alcohol use, steroid use for more than three months, body mass index, diagnosis of rheumatoid arthritis, osteoarthritis, chronic kidney disease, and liver disease), and quality of care indicators (documentation of the FRAX score, use of osteoporosis medications in patients with osteoporosis and high-risk osteopenia, and frequency of dual-energy X-ray absorptiometry (DXA) scans).

Results: A total of 462 patients were identified based on predetermined ICD-10 codes. After excluding 75 medical charts, the study sample consisted of 387 women: 225 (58.1%) with osteoporosis and 162 (41.9%) with osteopenia. Moreover, 321 (83%) were Hispanic or Latino. Risk factors included previous fracture in 46% (11.9%), glucocorticoid use for more than three months in 36 patients (9.3%), diagnosis of rheumatoid arthritis in 31 (8%), and smoking in 25 (6.5%). The FRAX score was documented for 145 patients (37.5%). Osteoporosis medication was prescribed to 151 patients (67.1%) with osteoporosis and to 40 patients (71.4%) with high-risk osteopenia. Among our study population, only 157 patients (40.6%) were taking vitamin D and calcium supplements.

Conclusion: Our study highlights care gaps in the assessment and management of osteoporosis/osteopenia among our Hispanic community. The most common risk factor identified was a history of previous fracture. Almost one-third of patients with osteoporosis and high-risk osteopenia did not receive pharmacological therapy. Most of our patients were not assessed for a 10-year fracture risk with the FRAX score and were not on vitamin D and calcium.

## Introduction

Around 10 million older adults in the United States are diagnosed with osteoporosis, with an even higher prevalence of osteopenia at about 43 million, according to the National Health and Nutrition Examination Survey (NHANES) survey in 2010. This prevalence varies within ethnic populations, with higher rates in the Mexican American population (13.4%), compared to the national average of 10.1%. The rate of fracture is also highest among Hispanic and White women [1,2].

The U.S. Preventive Services Task Force recommends initial screening for osteoporosis with bone measurement testing in women 65 years or older and in postmenopausal women younger than 65 years who are at increased risk of osteoporosis. This risk is determined by formal clinical risk assessment tools such as the osteoporosis self-assessment tool, osteoporosis risk assessment instrument, osteoporosis index of risk, simple calculated osteoporosis risk estimation, and Fracture Risk Assessment Tool (FRAX) tool [3]. Among these, the FRAX calculator is widely used due to its accessibility and strong validation across populations [4]. It incorporates age, weight, height, femoral neck bone mineral density (BMD), prior fracture, parental history of hip fracture, current smoking, glucocorticoid use, rheumatoid arthritis, secondary causes of osteoporosis, and alcohol intake to estimate a patient’s 10-year probability of major osteoporotic and hip fractures [4].

Bone densitometry, known as dual-energy X-ray absorptiometry (DXA), is used to evaluate the BMD. Despite the availability of DXA, osteoporosis frequently goes undiagnosed until fragility fractures occur [5]. Furthermore, treatment for osteoporosis is frequently underutilized. Many patients fail to receive appropriate pharmacological treatment due to concerns about long-term efficacy, potential side effects, or because treatment is never initiated by physicians [6,7]. Study suggested that less than 35% of patients diagnosed with osteoporosis are started on therapy, and fewer than 45% of these patients continue medication within six months of screening [8].

Given the lack of published data on osteoporosis treatment disparities and risk factors in our Hispanic community, this study aimed to determine osteoporosis risk factors presented in the study population and evaluate whether the osteoporosis screening and treatments have been underutilized in the community served by our clinic.

## Materials and methods

The present study is a retrospective chart review of female patients aged 50 years and older diagnosed with osteopenia/osteoporosis, who were seen at the Texas Tech University Health Sciences Center (TTUHSC) physician clinics in El Paso during the period of January 1, 2023, to December 31, 2023. Data were extracted from medical charts with the ICD-10 (10th revision of the International Classification of Diseases) codes for osteoporosis and osteopenia (M81.0, M81.8, M80, and M85.80).

The purposes of this research were to describe the osteoporosis risk factors among the study population and to evaluate the use of osteoporosis medications and the FRAX tool. Excluded criteria included charts without ICD-10 diagnostic codes M81.0, M81.8, M80, and M85.80, charts of female patients under 50 years of age, and charts of male patients. In addition, patients whose charts could not be located and charts lacking data beyond the ICD diagnosis code were excluded. Duplicate charts were also identified and removed. 

Institutional Review Board (IRB) approval was obtained from the Texas Tech University Health Sciences Center (TTUHSC), El Paso (approval no. E24108). Data were collected and analyzed using Research Electronic Data Capture (REDCap) hosted at TTUHSC El Paso. A standardized collection form was created by the research team (see Appendix). 

The following variables were collected: demographic information (self-reported race/ethnicity and age at the time of diagnosis), risk factors (previous fractures, parent fractured hip, current smoking, consumption of three or more units of alcohol daily, steroid use for more than three months, body mass index in kg/m², diagnosis of rheumatoid arthritis), DXA scan (femoral neck bone mineral density in g/cm2 of the first DXA), laboratory values (vitamin D level in ng/ml), quality of care measurement (documentation of FRAX fracture risk assessment tool, documentation of DXA scan, use of osteoporosis medications in patients with osteoporosis and high-risk osteopenia (defined as T-score between -1.0 and -2.5 with a risk of major osteoporotic fracture equal to or greater than 20% or a risk for hip fracture equal to or greater than 3% according to the FRAX calculator), and frequency of DXA BMD scans), medications (proton pump inhibitors, anti-epileptic drugs, aromatase inhibitors, sodium-glucose cotransporter 2 inhibitors, denosumab, teriparatide, romosozumab, selective estrogen receptor modulators, calcium supplements, and vitamin D), duration of bisphosphonate treatment, documented comorbid conditions (osteogenesis imperfecta, hyperthyroidism, hyperparathyroidism, type 1 diabetes, hypogonadism, premature menopause, and malabsorption syndromes, chronic kidney disease).

Variables were summarized using counts and percentages for categorical variables and mean ± standard deviation (SD) for continuous variables.

## Results

A total of 462 patients were identified based on predetermined ICD-10 codes. After excluding 75 medical charts due to age, sex, duplication, or missing information beyond the ICD-10 codes, 387 women were included in the study. Among these, 225 (58.1%) had a diagnosis of osteoporosis and 162 (41.9%) had osteopenia. The mean age at diagnosis was 69.9 years for patients with osteoporosis and 66.9 years for those with osteopenia.

**Figure 1:**
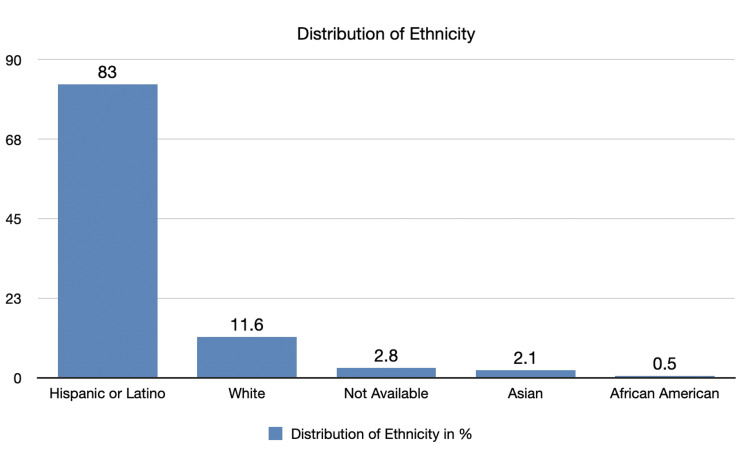
Distribution of self-reported race/ethnicity among the study population in percentage

Among 387 patients, 321 patients (83%) were Hispanic or Latino, 45 (11.6%) non-Hispanic White, eight (2.1%) non-Hispanic Asian, and two (0.5%) non-Hispanic Black or African American. Race/ethnicity data were not available in 11 (2.8%) medical charts (Figure 1). 

Risk factors used in the FRAX score calculator were assessed. Parental history of hip fracture was not documented in any of the medical charts across the cohort. Regarding smoking history, 25 (6.5%) were current smokers, 351 (90.7%) were non-smokers, and 11 (2.8%) had unknown smoking status. Five patients (1.3%) reported alcohol use, and 88 (22.7%) had no documentation of alcohol status. A total of 31 patients (8%) had a diagnosis of rheumatoid arthritis. Thirty-six patients (9.3%) had used glucocorticoids for more than three months (Table 1). The mean body mass index (BMI) is 28.5 kg/m² (Table 2). 

**Table 1:** Clinical risk factors N = number of patients

Variables	N (%)
Previous fracture	46 (11.9%)
Smoking history	25 (6.5%)
Glucocorticoid use	36 (9.3%)
Rheumatoid arthritis	31 (8.0%)
Alcohol use	5 (1.3%)

**Table 2:** Mean body mass index in kg/ m² N = number of patients. SD = standard deviation

	N	Mean	SD
Osteoporosis	221	27.0	5.58
Osteopenia	162	30.5	6.39
Overall	383	28.5	6.17

Medical conditions related to osteoporosis were recorded. Liver diseases were found in 26 patients (6.7%). These include hepatitis, cirrhosis, nonalcoholic liver disease, or any other type of liver disease. Fifteen patients (3.9%) had the diagnosis of hyperparathyroidism. Most of our patients did not have chronic kidney disease (94.1%). Only two patients (0.5%) were classified as having end-stage kidney disease. Malabsorption conditions collected in our study involve celiac disease, cystic fibrosis, Crohn’s disease, ulcerative colitis, status post-gastric resection or bypass, and were identified in three patients (0.78%). Hyperthyroidism, hypogonadism, premature menopause (less than 45 years), and osteogenesis imperfecta were documented in 0.2-1.3 % of the patients (Table 3). No case of type 1 diabetes was reported. 

**Table 3:** Documented comorbidities N = number of patients, CKD = chronic kidney disease

Comorbidities	Total		Osteoporosis		Osteopenia	
	N	%	N	%	N	%
Osteogenesis imperfecta	1	0.26	0	0	1	0.62
Hyperthyroidism	4	1.03	2	0.89	2	1.23
Hypogonadism	2	0.52	2	0.89	0	0
Premature menopause	5	1.29	3	1.33	2	1.23
Malabsorption	3	0.78	0	0	3	1.85
Liver disease	26	6.72	8	3.56	18	11.11
CKD	23	5.94	14	6.22	9	5.56

Regarding medications related to bone loss and/or increased fracture risk, 130 patients (33.6%) were taking proton pump inhibitors (PPI), 13 (3.4%) were using aromatase inhibitors, 21 (5.4%) were prescribed sodium-glucose cotransporter 2 inhibitors (SGLT2i), four (1.03%) were on antiepileptic medications, and two (0.5%) were taking selective estrogen receptor modulators (Table 4). 

**Table 4:** Medications associated with bone loss and/or increased fracture risk N = number of patients, SGLT2i = sodium–glucose cotransporter 2 inhibitors

Medications	Total		Osteoporosis		Osteopenia	
	N	%	N	%	N	%
Proton pump inhibitors	130	33.59	70	31.11	60	37.04
Antiepileptic	4	1.03	1	0.44	3	1.85
Aromatase inhibitors	13	3.36	6	2.67	7	4.32
SGLT2i	21	5.43	10	4.44	11	6.79

In terms of osteoporosis and osteopenia evaluation, the FRAX score was documented for 145 patients (37.5%). Among patients with osteopenia, 101 (62.3%) had a calculated FRAX score. A DXA scan was documented in 281 charts (72.6%). The mean age at the first DXA scan was 67.7 years. The second DXA was not available in 272 (70.2%) of the medical charts (Table 5). The interval between the first and second DXA scan was one to two years in 43 patients (11.1%), more than two to three years in 21 patients (5.4%), more than three to four years in 16 patients (4.1%), and more than four years in 33 patients (8.5%). We observed that the mean femoral neck BMD in the first DXA was 0.61 g/cm^2^ (Table 6).

**Table 5:** Dual-energy X-ray absorptiometry (DXA) results N = number of patients

Variables	Items	N
First DXA	Normal	11
	Osteopenia	162
	Osteoporosis	121
	Not available	93
Second DXA	Normal	0
	Osteopenia	66
	Osteoporosis	49
	Not available	272

**Table 6:** Age at first DXA, femoral neck BMD, and vitamin D level DXA = dual-energy X-ray absorptiometry, BMD = bone mineral density, N = number of patients, SD = standard deviation

	Total			Osteoporosis			Osteopenia		
	N	Mean	SD	N	Mean	SD	N	Mean	SD
Age at first DXA	358	67.7	7.48	207	68.83	8.62	152	66.2	7.48
Femoral neck BMD	271	0.61	0.31	140	0.57	0.34	131	0.66	0.28
Vitamin D level	70	39.40	17.06	39	41.64	18.13	31	36.57	15.45

With regard to treatment, osteoporosis medications were prescribed for 151 patients with osteoporosis (67.1%). These medications were taken by 40 of the 56 patients with high-risk osteopenia (71.4%). When combining patients with osteoporosis and those with high-risk osteopenia, a total of 191 patients (68%) were prescribed osteoporosis medications. Bisphosphonates were prescribed to 174 patients (45%) of our cohort. 14 were taking denosumab, two were using teriparatide, and one was on romosozumab. Among the group of patients taking bisphosphonate, 143 had been on this medication for less than three years. Four patients had been on bisphosphonates for more than five years. Calcium and vitamin D supplementation was documented in 157 of our cohort (40.6%), including 84 patients (37.3%) with osteoporosis and 73 (45.1%) with osteopenia (Table 7). As shown in Table 6, the vitamin D level was documented in 70 medical charts of our cohort, and the mean level was 39.40 ng/ml. 

**Table 7:** Number and percentage of patients with osteoporosis and osteopenia taking calcium, vitamin D, or both supplements N = number of patients

	Total		Osteoporosis		Osteopenia	
	N	%	N	%	N	%
Calcium	163	42.11	89	39.56	74	42.12
Vitamin D	212	54.78	118	52.44	94	54.78
Calcium and vitamin D	157	40.57	84	37.33	73	40.57

## Discussion

Osteoporosis is a well-recognized condition associated with a high incidence of fragility fractures. Its prevalence varies by ethnicity, with Mexican American women experiencing higher rates compared to the national average, and fracture incidence being highest among Hispanic and White women [1,2]. Given these disparities, it is important to recognize that Hispanic populations may face unique barriers, including differences in healthcare access, socioeconomic factors, and cultural perceptions of bone health. Despite the widespread knowledge and availability of guidelines, there are still gaps in both screening and treatment of osteoporosis [9]. Our study identified several deficiencies in care, particularly regarding the use of the FRAX score, vitamin D and calcium supplementation, and pharmacological therapy for osteoporosis. These findings suggest ongoing challenges in the effective assessment and management of osteoporosis in our majority Hispanic community. 

All postmenopausal women should undergo an assessment of their 10-year fracture risk [10]. In the United States, the main tool used for calculating this risk is the FRAX score calculator. FRAX score is especially important for the evaluation of the indication of osteoporosis medications for patients with osteopenia. In our study, FRAX scores were not calculated for 61 patients (37.6%) with osteopenia and for 242 patients with osteoporosis and osteopenia (62.5%). This low adherence to current guidelines may be attributed to poor documentation in the medical records and the lack of immediate access to the FRAX calculator during clinical visits. In addition, limited integration of risk assessment tools within the electronic health record, time constraints during visits, and insufficient provider training may also contribute. A potential solution is to integrate the FRAX calculator into the electronic medical record system, implement automated prompts within clinical workflows, and provide targeted provider education.

The FRAX score calculator incorporates multiple risk factors, including age, sex, weight, height, previous fracture, parental history of hip fracture, current smoking, long-term glucocorticoid use, rheumatoid arthritis, secondary causes of osteoporosis, and alcohol consumption [4]. In our study, previous fracture was documented in 46 (11.9%), glucocorticoid use for more than three months in 36 patients (9.3%), and diagnosis of rheumatoid arthritis in 31 patients (8%). Other risk factors were less frequently reported. Secondary causes of osteoporosis were also uncommon in our study (Table 3). Our findings are consistent with a previous study that has identified prior fracture as the most common risk factor among middle-aged and older adults [11]. 

Osteoporosis and osteopenia are diagnosed using DXA. This scan is recommended for all women 65 years or older. The mean age for the first DXA in our population was approximately 68 years. For women under 65, the decision regarding imaging screening for osteoporosis is based on the patient’s risk factors [10]. DXA should be repeated every one to three years, depending on the clinical context [12]. However, the timing of repeat scans was often undocumented in our study. Only 64 patients (16.5%) underwent a repeat DXA between one and three years after the initial screening, and 33 patients (8.5%) had a repeat scan more than four years. These findings should be interpreted with caution, given the short study period and the possibility that some patients were recently diagnosed during the study timeframe.

The American Association of Clinical Endocrinologists recommends maintaining 25-hydroxyvitamin D levels at or above 30 ng/ml for patients with osteoporosis [10]. In our study, the mean vitamin D level of patients with osteoporosis was 41.64 ng/ml. A daily intake of 1200 mg of calcium and 800-1000 IU of vitamin D is advised by the National Osteoporosis Foundation [13]. Among our study population, 212 patients (54.8%) were taking vitamin D, and 163 (42.1%) were taking calcium supplements. Only 157 patients with osteoporosis or osteopenia (40.6%) were taking both calcium and vitamin D supplementation. In addition to supplementation, all patients with osteoporosis and osteopenia should also maintain regular exercise, avoid smoking, and excessive alcohol consumption [13]. We did not collect data on the dose of vitamin D and calcium, nor on lifestyle modifications. 

Pharmacological therapy is indicated for patients with osteoporosis, defined by a T-score of -2.5 or lower. It is also recommended for patients with high-risk osteopenia (T-score between -1.0 and -2.5) who have a major osteoporotic fracture or more equal 20% or a risk of hip fracture of more or equal 3% [10]. Bisphosphonates remain the initial pharmacological treatment for most patients with primary osteoporosis and high-risk osteopenia. Denosumab, a RANK ligand inhibitor, serves as an alternative for patients who have contraindications to bisphosphonates or experience adverse effects with them. The treatment with recombinant PTH (teriparatide) or the sclerostin inhibitor (romosozumab) is reserved for patients with osteoporosis at very high risk for fracture [14].

In our study, 67.1% of patients with osteoporosis were receiving pharmacological treatment. When considering both osteoporosis and high-risk osteopenia, 191 patients (68%) were on osteoporosis medications. Specifically, 174 patients were on bisphosphonates, 14 were on denosumab, two were on Teriparatide, and one was on romosozumab. These data highlight a significant gap in treatment, with 32% of eligible patients not receiving pharmacological therapy. This rate of undertreatment is higher than that reported in previous studies, which found that approximately 21 to 24% of women with osteoporosis or recent fractures were not receiving appropriate medications [15,16]. The treatment gap observed in our study may be related to medication side effects, cost, low adherence among patients, or lack of timely treatment initiation by physicians. Strategies to reduce disparities in treatment include targeted education for healthcare providers and increased patient awareness about the importance of osteoporosis therapy. 

There are several limitations to our study. As a retrospective chart review, our study is affected by missing or incomplete data due to reliance on clinical documentation. The absence of documented FRAX scores or initiation of osteoporosis medications and vitamin D and calcium supplementation may not necessarily reflect a true gap in care. It may instead be due to poor documentation or clinical decisions not recorded in the chart. We also did not assess the reasons for the underuse of FRAX scores, vitamin D, and calcium supplementation, or pharmacologic treatment. In addition, the risk factors identified in our study may not accurately represent those of other similar populations. Lifestyle modifications, such as diet and exercise, were not collected in our study. Lastly, we did not compare outcomes between Hispanic and non-Hispanic patients, so differences among ethnic groups could not be documented. However, cultural and socioeconomic factors may influence osteoporosis care in Hispanic women. Language barriers, limited health literacy, and restricted access to healthcare and financial resources may contribute to lower adherence to osteoporosis screening recommendations and long-term therapy. 

## Conclusions

Our study demonstrated significant gaps in the assessment and management of osteoporosis and osteopenia among women in our predominantly Hispanic population. The documentation and use of the FRAX score, as well as repeat DXA scan within the recommended one- to three-year interval, were suboptimal. In addition, a considerable proportion of eligible patients were not receiving appropriate pharmacologic therapy or supplementation with vitamin D and calcium, despite established guidelines. Among the risk factors analyzed, history of previous fracture and glucocorticoid use were the most documented.

In summary, this retrospective chart review highlights the need to improve adherence to osteoporosis screening and treatment protocols in routine practice. Healthcare systems should integrate risk-assessment tools and screening reminders within electronic health records and expand provider- and patient-education programs about osteoporosis and osteopenia. Further study may be warranted to evaluate the specific risk factors and barriers contributing to the gap in care among Hispanic populations.

## Figures and Tables

**Figure 2:**
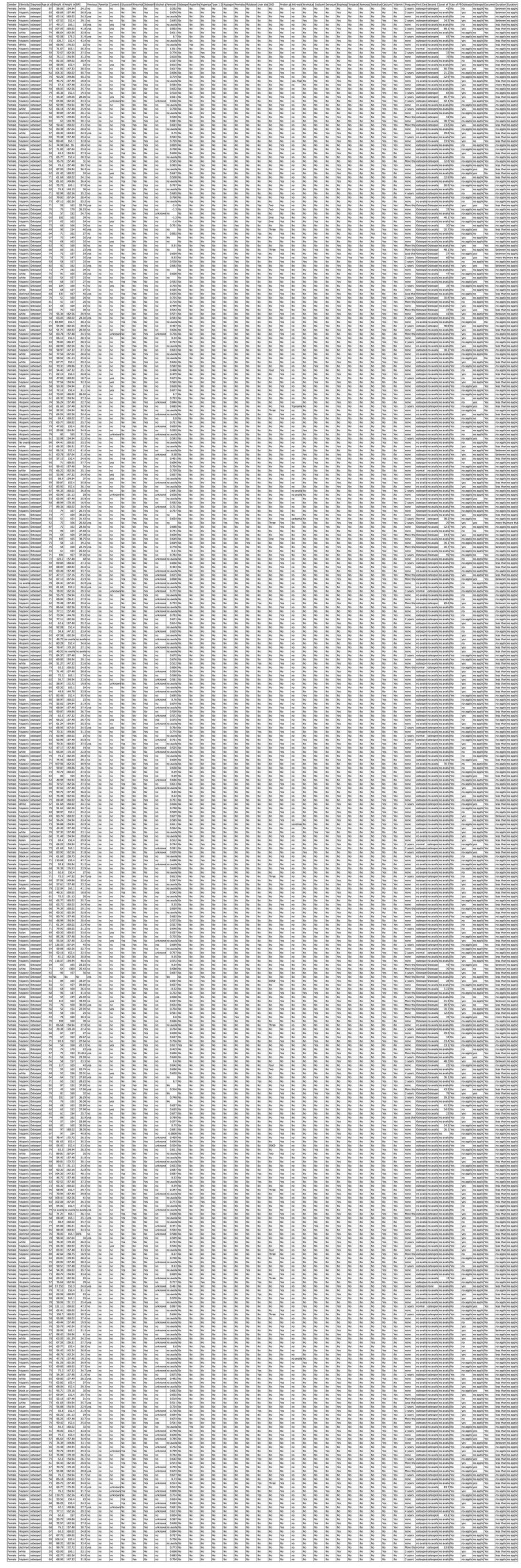
Collection form

## Appendices

Standardized collection form
